# Supplementary material for: Unveiling the Lipid Features and Valorization Potential of Atlantic Salmon (Salmo salar) Heads
Source: Mar Drugs. 2024 Nov 15;22(11):518. doi: 10.3390/md22110518 (PMC11595946; doi:10.3390/md22110518)
Supplement: Supplementary file 1 [file marinedrugs-22-00518-s001.zip › Supplementary Figures.pdf]

Unveiling the Lipid Features and Valorization Potential of Atlantic Salmon (Salmo salar) Heads

João Pedro Monteiro 1,2,3,* , Tiago Sousa 1,2 , Tânia Melo 1,2 , Carla Pires 4,5 , António Marques 4,5 , Maria Leonor Nunes 5 , Ricardo Calado 6,* and M. Rosário Domingues 1,2,*

1 Centro de Espetrometria de Massa & LAQV-REQUIMTE & Departamento de Química, Universidade de Aveiro, Campus Universitário de Santiago, 3810-193 Aveiro, Portugal; tmms@ua.pt (T.S.); taniamelo@ua.pt (T.M.)

2 CESAM & Departamento de Química, Universidade de Aveiro, Campus Universitário de Santiago, 3810-193 Aveiro, Portugal

3 CIVG—Vasco da Gama Research Center/EUVG—Vasco da Gama University School, 3020-210 Coimbra, Portugal

4 Division of Aquaculture, Upgrading and Bioprospection, Portuguese Institute for the Sea and Atmosphere (IPMA, I.P.), Av. Doutor Alfredo Magalhães Ramalho 6, 1495-165 Algés, Portugal; cpires@ipma.pt (C.P.); amarques@ipma.pt (A.M.)

5 Interdisciplinary Centre of Marine and Environmental Research (CIIMAR/CIMAR-LA), University of Porto, Terminal de Cruzeiros do Porto de Leixões, Av. General Norton de Matos s/n, 4450-208 Matosinhos, Portugal; nunes.leonor@gmail.com

6 ECOMARE & CESAM & Departamento de Biologia, Universidade de Aveiro, Campus Universitário de Santiago, 3810-193 Aveiro, Portugal

* Correspondence: jpspmonteiro@yahoo.com (J.P.M.); rjcalado@ua.pt (R.C.); mrd@ua.pt (M.R.D.)

Supplementary Figures

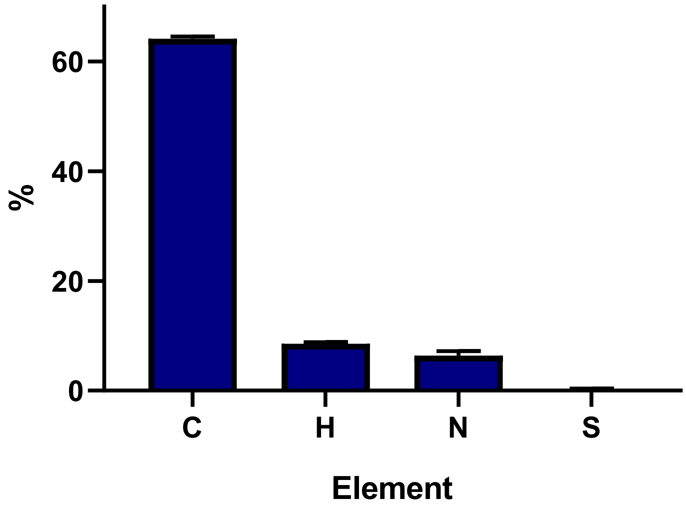

Supplementary Figure S1: Elemental composition of the grinded salmon head. Data are shown as mean ± standard deviation (SD) for 5 samples of grinded salmon heads (N=5).

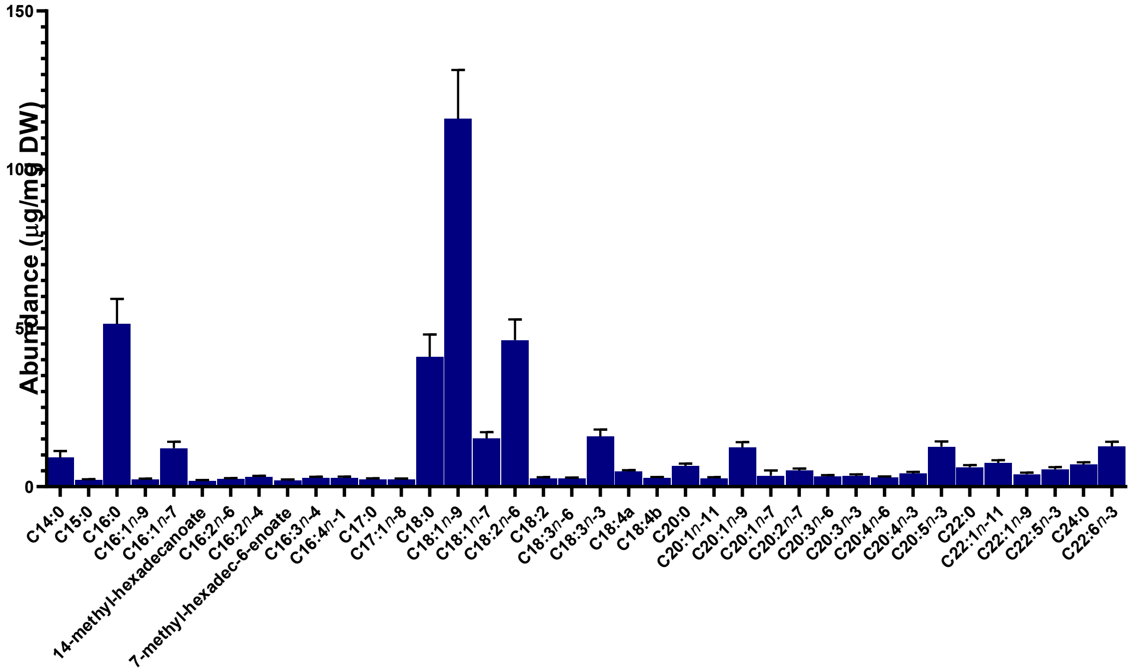

Supplementary Figure S2: Fatty acid profile of the grinded salmon heads as presented in absolute terms. Data are shown as mean ± standard deviation (SD) for 5 samples of grinded salmon heads (N=5).

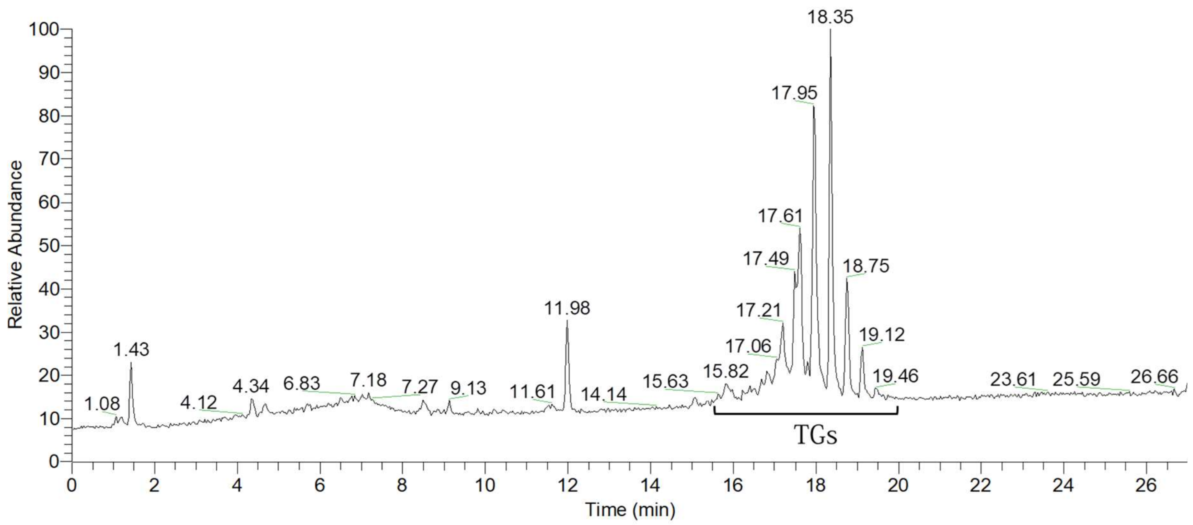

Supplementary Figure S3: Total ion count chromatogram of a grinded salmon sample in the positive mode.
